# Identification of genes regulating traits targeted for domestication of field cress (*Lepidium campestre*) as a biennial and perennial oilseed crop

**DOI:** 10.1186/s12863-018-0624-9

**Published:** 2018-05-29

**Authors:** Cecilia Gustafsson, Jakob Willforss, Fernando Lopes-Pinto, Rodomiro Ortiz, Mulatu Geleta

**Affiliations:** 1 Department of Plant Breeding, Swedish University of Agricultural Sciences, Box 101, SE-23053 Alnarp, Sweden; 2 Department of Plant Protection Biology, Swedish University of Agricultural Sciences, Box 102, SE-23053 Alnarp, Sweden; 3 Department of Animal Breeding and Genetics, Swedish University of Agricultural Sciences, Box 7023, SE-750 07 Uppsala, Sweden

**Keywords:** Domestication, Field cress, *Lepidium campestre*, Pod shattering, Vernalization

## Abstract

**Background:**

The changing climate and the desire to use renewable oil sources necessitate the development of new oilseed crops. Field cress (*Lepidium campestre*) is a species in the Brassicaceae family that has been targeted for domestication not only as an oilseed crop that produces seeds with a desirable industrial oil quality but also as a cover/catch crop that provides valuable ecosystem services. *Lepidium* is closely related to *Arabidopsis* and display significant proportions of syntenic regions in their genomes. *Arabidopsis* genes are among the most characterized genes in the plant kingdom and, hence, comparative genomics of *Lepidium-Arabidopsis* would facilitate the identification of *Lepidium* candidate genes regulating various desirable traits.

**Results:**

Homologues of 30 genes known to regulate vernalization, flowering time, pod shattering, oil content and quality in *Arabidopsis* were identified and partially characterized in *Lepidium*. Alignments of sequences representing field cress and two of its closely related perennial relatives: *L. heterophyllum* and *L. hirtum* revealed 243 polymorphic sites across the partial sequences of the 30 genes, of which 95 were within the predicted coding regions and 40 led to a change in amino acids of the target proteins. Within field cress, 34 polymorphic sites including nine non-synonymous substitutions were identified. The phylogenetic analysis of the data revealed that field cress is more closely related to *L. heterophyllum* than to *L. hirtum*.

**Conclusions:**

There is significant variation within and among *Lepidium* species within partial sequences of the 30 genes known to regulate traits targeted in the present study. The variation within these genes are potentially useful to speed-up the process of domesticating field cress as future oil crop. The phylogenetic relationship between the *Lepidium* species revealed in this study does not only shed some light on *Lepidium* genome evolution but also provides important information to develop efficient schemes for interspecific hybridization between different *Lepidium* species as part of the domestication efforts.

**Electronic supplementary material:**

The online version of this article (10.1186/s12863-018-0624-9) contains supplementary material, which is available to authorized users.

## Background

Domestication is the process of selecting genetic polymorphisms for traits that suit human needs. The “domestication syndrome” describes the main differences between domesticated and wild plants. Traits that normally are associated with the domestication syndrome include reduced seed dormancy, seed dispersal resistance, free threshing and high number of seeds per plant [1]. Historically, domestication of plants has been a slow and a labor-intense task as breeders have relied only on phenotypes to improve the crop. Although this approach has been very successful, it has its own drawbacks. It is time consuming, costly and can be influenced by the environment. The recent rapid development of genomics can make it possible to domesticate a new plant with less labor and within a significantly shorter time frame. The use of genomic tools and resources can speed up a domestication process as breeders can select plants bearing desirable traits before the traits have been expressed, using appropriate DNA markers and high-throughput precise phenotyping.

While farming systems with annual crops have provided us with unprecedented yield, they have also contributed to ecosystem problems such as soil erosion and water runoff [2]. Perennial crops normally have deeper root systems that can prevent soil erosion, reduce water runoff and nutrient leakage. In addition, perennial crops may require less herbicide treatment as its extended growing season enables it to outcompete weeds. The few examples of successful breeding programs of perennial crops include perennial rice and intermediate wheatgrass [3, 4]. There are also currently interesting attempts to develop perennial oil crops such as sunflower [5].

To meet the growing demand for a renewable oil production and reduce the negative effects of annual crop systems, we are in the process of domesticating a new seed oil crop: field cress (*Lepidium campestre* (L.) R. Br., also called pepperworth), which is a wild species in the Brassicaceae family. It has many good agronomic characteristics, which makes it a promising candidate for domestication. It has a relatively small diploid genome with a sporophytic chromosome number of 2n =16 [6]. Field cress is a self-fertilized biennial plant with closely related perennials (*L. hirtum* (L.) Sm. and *L. heterophyllum Benth.*), which can be used as a source of perenniality genes for the development of perennial field cress [7]. Although polyploids are common among *Lepidium* species, both *L. heterophyllum* and *L. hirtum* are diploids with 2n =16 chromosomes and can be cross-hybridized with field cress to produce viable hybrid plants [8]. Moreover, field cress is extremely winter hardy, resistant to the pollen beetle, have a high seed yield (> 4500 kg ha^− 1^) and a good seed size (half the size of that of large seeded rapeseed) [9, 10]. Field cress produces a high-quality seed oil suitable for industrial use, with linolenic and erucic acids being the main components.

Field cress has been targeted for domestication as an under sown cover-, catch- and oilseed crop to prevent nutrient leaching as well as being a high yielding crop [10]. It can be under sown with a spring cereal and serve as a cover and catch crop, after the harvest of the cereal in the summer, and finally harvested as an oil crop the second year. Hence, field cress holds high agronomic potential and eco-friendliness as it could simultaneously function as a high yielding oil crop and a cover/catch crop. Unlike rapeseed, which is not widely cultivated in areas with strong winter due to its poor winter-hardiness, field cress has been proven to be productive in a colder climate. Thus, cultivation of field cress could be of great value to farmers in the northern parts of temperate regions, e.g. Nordic Europe. Both crossbreeding and genetic engineering based approaches are being used for domestication of field cress. In line with this, floral dip based-, and tissue culture based transformation protocol have been successfully established for field cress [11, 12]. However, as the use of genetically modified plants is still very restricted within Europe, crossbreeding techniques are still essential.

As the cost for sequencing technology has been greatly reduced, comparative genomics has become an increasingly important tool in agriculture. *Arabidopsis thaliana* (thale cress) is among the most investigated plants in the plant kingdom and since the publication of the genome in the year 2000 [13], a vast number of genes have been identified and characterized. The publication of 1135 additional *A. thaliana* genomes provided information about the global pattern of polymorphisms [14]. Seed dispersal resistance, flowering time, winter hardiness, perenniality, oil content and oil quality are among the major traits targeted for field cress breeding at early stages of the domestication process. A number of genes regulating these traits are well known in *Arabidopsis* and *Brassica*, which are in the same Brassicaceae family as field cress. The close evolutionary relationship between *Arabidopsis* and field cress makes *Arabidopsis* an ideal species to identify orthologs of these genes in field cress through comparative genomics. Analyses of genes that are associated with vernalization and flowering time is important in order to facilitate the development of a winter hardy perennial field cress. *MADS*-box genes such as *FLOWERING LOCUS C* (*FLC*) play a key role in regulating plant developmental responses to temperature [15, 16]. The gene *FRIGIDA* (*FRI*) enhances the expression of *FLC* and has been demonstrated to be of great importance for vernalization and flowering time [17, 18].

*VERNALIZATION INSENSITIVE 3* (*VIN3*) is part of the polycomb repression complex PRC2 which establishes repression of *FLC* and is one of the earliest responses to vernalization [19, 20]. Downregulation of *FLC* promotes floral development and the low levels of *FLC* are maintained after the cold treatment by *REDUCED VERNALIZATION RESPONSE 1* and *2* (*VRN1* and *VRN2*) which serves as a molecular memory of the vernalization [20, 21]. *SUPRESSOR OF OVEREXPRESSION OF CO1* (*SOC1*, also *AGAMOUS LIKE 20*/*AGL20*) and *FRUITFULL* (*FUL*) are controlling flowering as well as determinacy of meristems but also seem to be involved in inhibiting longevity in annual plants [22–24]. *FLC*, *FRI*, *VIN3*, *VRN1* and *VRN2* were among main targets in the present study as their role in vernalization and flowering time has been well established. *SOC1* and *FUL* were also interesting for their reported effect on perenniality. Analysis of field cress orthologs of *AGAMOUS LIKE 6* (*AGL6*)*, AGAMOUS LIKE 16* (*AGL16*) *and MADS AFFECTING FLOWERING 2–5* (*MAF2–5*) which are reported to have effect on these traits [25–28] are also of interest.

Shattering of pods (fruit dehiscence) can lead to significant yield losses and pod shattering resistance is therefore a highly advantageous trait in any domesticated crop. Hence, identification and analysis of transcription factor genes, *FUL* and *REPLUMLESS* (*RPL*), together with the valve identity genes *INDEHISCENT* (*IND*), *ALCATRAZ* (*ALC*), *SHATTERPROOF 1* and *2* (*SHP1* and *SHP2*) that are responsible for the establishment of the valve margin in the seed-containing pod, and regulate thereby the release of seeds from the pod, [29–34] should be targeted as important steps towards the development of shatter proof field cress cultivars. In addition, genes encodin*g ARABIDOPSIS DEHISCENCE ZONE POLYGALACTURONASE 1* and *2* (*ADPG1* and *ADPG2*) and the *NAC DOMAIN CONTAINING PROTEIN 12* (*NAC012*/*SND1*) which are contributing to fruit dehiscence [35, 36] are also important targets.

Because field cress is targeted as an oilseed crop, genes that code for both oil content and quality need to be analyzed as integral part of the domestication process so that acceptable levels of oil content and quality can be achieved. Among the various genes involved in the biosynthesis of fatty acid *ACYL-COA:DIACYLGLYCEROL ACYLTRANSFERASE 1* (*TAG1*) and *WRINKLED 1* (*WRI1*) regulate oil production in many species, including *Arabidopsis* and *FATTY ACID DESATURASE 2* (*FAD2*), *FATTY ACID ELONGATION 1* (*FAE1*) and *3-KETOACYL-COA-SYNTHASE 8* (*KCS8*) are controlling seed oil composition in most oil crops [37–42]. Other important traits in field cress include host plant resistance to pathogens and seed dormancy. Hence, genes involved in regulating these traits are important targets. Homologous sequences in field cress for host plant resistance gene *FERONIA* (*FER*) and plant defense gene *AUTOPHAGY RELATED 5* (*ATG5*), genes that are involved in germination of seeds *AGAMOUS-LIKE 11* (*AGL11*) and *HIGHLY ABA-INDUCED PP2C PROTEIN 2* (*HAI2*/*AIP1*) [43–46] were also in focus.

The present study aimed at the identification of the orthologues of the aforementioned genes in field cress through comparative analysis of its genome with that of *Arabidopsis* and *Brassica*; characterization of the genetic diversity of these genes in field cress; and analyzing the effect of different mutations in each gene on the plant phenotypes in field cress and related species.

## Methods

### Plant material

A total of 31 individual plants of field cress, *L. hirtum, L. heterophyllum* and interspecific hybrids of field cress and *L. heterophyllum* (CHe hybrids) was used for this study (Table 1). Ten field cress and two F_3_ CHe hybrids were among 18 *Lepidium* samples used for sequencing the restriction site associated DNA (RAD) [46]. The remaining 19 individuals –comprising 14 field cress, three *L. hirtum* and two *L. heterophyllum*– (Table 1) were used for the sequencing of partial sequences of target genes after they were identified based on comparative genomic analysis of *Lepidium* RAD-sequences and genomic sequences of *Arabidopsis* and *Brassica*. These *Lepidium* accessions were obtained from various genebanks and botanical gardens in Europe as well as after collecting populations from various regions in Sweden. Except the CHe hybrids, the samples were selfed at least for four generations in a greenhouse. The CHe hybrids are the result of interspecific hybridization followed by two rounds of selfing. These samples were chosen for their geographical spread and their phenotypic variation.

**Table 1:** Sample codes, source and country of origin of different genotypes of three *Lepidium* species and CHe hybrids analyzed for genetic variation within partial sequences of various genes regulating desirable traits. *L. hirtum* is represented by three subspecies. Genotypes 1–14 and 27–31 were amplified and sequenced using newly designed primers (Additional file 1: Table S1) while genotypes 15–26 were those used in the RAD-Sequencing project

No	Sample code	Species	Source accession/population		Country of origin
			name	obtained from	
1	LcSma	*L. campestre*	Mörbylånga	Newly collected	Öland, Sweden
2	LcSstu	*L. campestre*	Stuvsta	Newly collected	Södermanland, Sweden
3	LcCze	*L. campestre*	PI 633248	USDA-ARS	Czechoslovakia
4	LcGer1	*L. campestre*	LEP 122	IPK, Germany	Germany
5	LcSho	*L. campestre*	Höör2	Newly collected	Skåne, Sweden
6	LcGer2	*L. campestre*	LEP 93	IPK, Germany	Germany
7	LcSlj	*L. campestre*	Ljugarn	Newly collected	Gotland, Sweden
8	LcGer3	*L. campestre*	PI 633251	USDA-ARS	Germany
9	LcSkr	*L. campestre*	Kristianstad	Newly collected	Skåne, Sweden
10	LcSar	*L. campestre*	Årsta1	Newly collected	Södermanland, Sweden
11	LcSvi	*L. campestre*	Viken	Newly collected	Skåne, Sweden
12	LcSsk	*L. campestre*	Skövde	Newly collected	Västergötland, Sweden
13	LcSsp	*L. campestre*	Spjutstorp	Newly collected	Skåne, Sweden
14	LcSve	*L. campestre*	Ventlinge	Newly collected	Öland, Sweden
15	LcFra	*L. campestre*	PI 633252	USDA-ARS	France
16	LcGre1	*L. campestre*	LEP 89	IPK, Germany	Greece
17	LcSst	*L. campestre*	094–10	Newly collected	Stjärnelund, Sweden
18	LcUK	*L. campestre*	0018580	Royal Botanic Garden, UK	?
19	LcSga	*L. campestre*	Gävle	Newly collected	Gävleborg, Sweden
20	LcStr	*L. campestre*	Trelleborg	Newly collected	Skåne, Sweden
21	LcGer4	*L. campestre*	LEP 94	IPK, Germany	Germany
22	LcGre2	*L. campestre*	LEP 92	IPK, Germany	Greece
23	LcDen1	*L. campestre*	4932 4	Botanic Garden-Denmark	?
24	LcDen2	*L. campestre*	NGB22634	NordGen, Sweden	Denmark
25	CH1^a^	*L. campestre x heterophyllum* ^a^	LEP 89 & 597856	IPK, Germany & USDA-ARS	Germany & Spain
26	CH2^a^	*L. campestre x heterophyllum* ^a^	Huddinge & 597856	Newly collected & USDA-ARS	Sweden & Spain
27	LheGer	*L. heterophyllum*	1988/690–148	Marburg Bot. garden, Germany	?
28	LheSha	*L. heterophyllum*	Hästveda	Newly collected	Skåne, Sweden
29	LhiIta	*L. hirtum* ssp. *nebrodense*	PI633253	USDA-ARS	Italy
30	LhiSpa	*L. hirtum* ssp. *calycotrichum*	PI597858	USDA-ARS	Spain
31	LhiMor	*L. hirtum* ssp. *atlanticum*	Ames 21387	USDA-ARS	Morocco

^a^hybrid of *L. campestre* and *L. heterophyllum*;? = no information

### Comparative genomics/bioinformatics and primer design

The RAD-sequencing conducted on 18 *Lepidium* samples comprising 10 field cress, two F_3_ CHe hybrids and three progenies of each of the two CHe hybrids at Edinburgh Genomics (School of Biological Sciences, University of Edinburgh, EH9 3FL, Edinburgh, UK) produced over 190,000 consensus RAD-sequences [47]. A BLAST search for sequences that match the RAD sequences in the National Center for Biotechnology Information (NCBI) database identified sequences that have 75 to 90% sequence identity with the *Arabidopsis* genome sequences, with e-values ranging from 0.0 to 1e^− 11^. Additional top hits included sequences of other Brassicaceae species such as *Brassica rapa, Camelina sativa* and *Boechera stricta*. All DNA sequences used for comparative genomic analysis except those of *Lepidium* were retrieved from the NCBI database.

DNA sequences of 30 genes (see Table 2) from *A. thaliana* and field cress known to be regulators of desirable traits for plant domestication were used as query sequences to find any homologous sequences in the RAD sequence pool. However, since the RAD-sequences were short sequences (117 nucleotides (nt) to 567 nt long), they only represent short segments of these genes. In total, DNA sequences homologous to partial sequences of 24 different genes were identified through using this approach (Additional file 1: Table S1). Primers were then designed using field cress RAD-sequences as a template by mainly targeting coding regions in 18 of these 24 genes. Although sequences homologous to partial sequences of the remaining six genes (*AGL11*, *ALC*, *FUL, HAI2, IND,* and *SOC1*) were found among the RAD-sequences, conserved *Arabidopsis* sequences were used to design primers in order to expand the regions to be sequenced. Six of the 30 genes (*ADPG1*, *AP2, FAE1, FLC, NAC012* and *RPL*) did not show any homology to the RAD sequences, and hence conserved sequences in *A. thaliana* or previously published field cress mRNA sequences were used as a template to design primers for amplification of orthologous regions in field cress.

**Table 2:** Sequence identity (%) between field cress and seven other Brassicaceae species within partial sequences of coding regions of 30 genes regulating desirable traits in crops

Gene	Trait/gene function	*A.lyrata*	*A. thaliana*	*B.rapa*	*B.napus*	*B.oleracea*	*Camelina sativa*	*Capsella rubella*	Mean
*AGL11*	SD	95	95	91	92	92	96	96	93.9
*SOC1*	FT	94	94	93	93	93	94	93	93.4
*TAG1*	OC	94	93	93	93	93	93	95	93.4
*AGL6*	FT	95	98	90	91	91	92	92	92.7
*VRN1*	VRN	94	94	90	90	90	92	94	92.0
*NAC012*	PSH	92	92	91	91	90	93	92	91.6
*FER*	DR	93	93	89	89	89	92	91	90.9
*FUL*	FT, PSH	92	92	89	89	89	90	90	90.1
*FLC*	FT, VRN	89	90	88	88	88	90	92	89.3
*MAF2*	VRN	93	95	87	84	85	91	89	89.1
*MAF5*	VRN	93	95	87	84	85	91	89	89.1
*AP2*	FT	89	89	89	89	89	90	88	89.0
*ADPG1*	PSH	89	91	88	86	87	91	90	88.9
*SHP1*	PSH	90	90	89	89	89	89	86	88.9
*SHP2*	PSH	91	89	86	87	87	89	92	88.7
*FAD2*	OQ	91	91	85	85	85	90	91	88.3
*VRN2*	VRN	92	90	83	84	84	89	88	87.1
*GTR2*	GTR	88	88	86	86	86	88	88	87.1
*FAE1*	OQ	89	88	85	85	85	89	88	87.0
*RPL*	FT, PSH	89	89	85	85	84	88	88	86.9
*VIN3*	VRN	87	86	85	85	84	85	85	85.3
*WRI1*	OC	88	88	82	82	83	86	87	85.1
*KCS8*	OQ	88	87	82	83	82	87	86	85.0
*ADPG2*	PSH	85	84	82	82	82	89	90	84.9
*IND*	PSH	86	85	84	86	76	85	84	83.7
*ALC*	PSH	82	83	81	81	81	82	84	82.0
*AGL16*	FT	82	84	81	81	82	82	81	81.9
*FRI*	VRN, FT	84	81	81	81	80	82	83	81.7
*HAI2*	SD	82	82	81	80	80	81	82	81.1
*ATG5*	PD	82	81	79	80	72	84	80	79.7
Mean		89.3	89.2	86.1	86.0	85.4	88.7	88.5	87.6

*DR* Disease resistance, *FT* Flowering time, *OC* Oil content, *OQ* Oil quality, *PD* Plant defense, *PSH* Pod shattering, *SD* Seed dormancy, *GTR* Glucosinolate Transport, *VRN* Vernalization. Note: The matching of the *Lepidium* sequences with the right *Arabidopsis gene sequences* is highly significant, with e-values ranging from 0.0 to 1e^−11^

### DNA extraction

Genomic DNA was obtained by sampling leaf tissue from young plants, grown in a green house, which was flash-frozen in liquid nitrogen and homogenized by vigorously shaking in a Retsch MM400. The samples were incubated with 1 ml of pre-warmed CTAB buffer (0.1 M Tris, 20 mM EDTA, 1.4 M NaCl, 2% CTAB, pH 7.5) for 1 h at 52 °C. The samples were then centrifuged for 15 min at 14.1 rpm in an Eppendorf miniSpin tabletop centrifuge. The supernatant was then transferred to sample plate and DNA was extracted using the Qiacube DNA extraction robotic workstation (Qiagen). The extracted DNA was finally ran on a 1% agarose gel and checked for impurities on a Nanodrop. DNA from the 19 individuals (Table 1) was used for screening partial sequences of the 30 target genes (Table 3).

**Table 3:** Variation (SNPs and indels) found in aligned sequences of *L. campestre*, *L. heterophyllum* and *L. hirtum* as well as in aligned sequences of different *L. campestre* genotypes. The analyzed sequence length, number of polymorphism (Polym), number of nonsynonymous mutations (Non-syn), the number of species specific polymorphisms (Species spec. polym) and percent polymorphism per nucleotide (Polym/nt) are listed according to gene

Gene	Trait/gene function	Analyzed sequence length^a^	*L. campestre + L. hirtum + L heterophyllum*				*L. campestre only*		
			Polym^a^	Non-syn^b^	Polym/nt^a^ (%)	Species spec. polym^a^,^c^	Polym^a^	Non-syn^b^	Polym/nt^a^ (%)
*FRI*	vernalization, flowering time	606_498	13_8	6	2.1_1.6	0_0 (0)	3_3	2	0.5_0.6
*MAF2*	vernalization	532_181	7_3	2	1.3_1.7	0_0 (0)	7_3	2	1.3_1.7
*MAF5*	vernalization	365_206	4_4	1^d^	1.1_1.9	0_1 (0)	1_1	0	0.3_0.5
*VIN3*	vernalization	1347_1121	7_5	4	0.5_0.4	0_1 (1)	0_0	0	0_0
*VRN1*	vernalization	1545_501	2_1	1	0.1_0.2	0_0 (0)	2_1	1	0.1_0.2
*VRN2*	vernalization	1405_536	3_1	0	0.2_0.2	0_1 (0)	0_0	0	0_0
*FLC*	vernalization, flowering time	840_170	17_5	2	2_2.9	9_5 (3)	0_0	0	0_0
*AGL6*	flowering time	570_182	2_0	0	0.4_0	1_0 (0)	0_0	0	0_0
*AGL16*	flowering time	772_182	9_1	1^d^	0.3_0	3_0 (1)	1_0	0	0.13_0
*AP2*	flowering time	750_447	9_4	2	1.2_0.9	0_4 (0)	0_0	0	0_0
*SOC1*	flowering time	797_290	6_1	1^d^	0.8_0.3	0_0 (0)	0_0	0	0_0
*FUL*	flowering time, pod shattering	942_295	3_0	0	0.3_0	1_0 (1)	0_0	0	0_0
*RPL*	flowering time, pod shattering	560_524	1_1	1	0.2_0.2	0_0 (0)	1_1	1	0.2_0.2
*ADPG1*	pod shattering	650_138	15_0	0	2.3_0	11_0 (2)	0_0	0	0_0
*ADPG2*	pod shattering	465_212	4_3	0	0.9_1.4	0_0 (0)	0_0	0	0_0
*ALC*	pod shattering	849_321	7_1	1	0.8_0.3	3_0 (2)	0_0	0	0_0
*IND*	pod shattering	1025_440	11_5	3	1.1_1.1	0_3 (2)	0_0	0	0_0
*NAC012*	pod shattering	400_305	1_0	0	0.3_0	0_0 (0)	0_0	0	0_0
*SHP1*	pod shattering	1112_330	19_5	0	1.7_1.5	0_0 (0)	11_3	0	1_0.9
*SHP2*	pod shattering	962_411	15_6	2	1.6_1.5	1_1 (1)	1_1	1	0.1_0.2
*FAD2*	oil quality	710_416	5_4	2	0.7_1	0_0 (0)	1_1	1	0.1_0.2
*FAE1*	oil quality	490_478	2_2	0	0.4_0.4	0_2 (0)	0_0	0	0_0
*KCS8*	oil quality	1382_1362	12_9	5	0.9_0.7	0_0 (0)	0_0	0	0_0
*TAG1*	oil content	962_414	15_5	0	1.6_0	1_0 (0)	1_0	0	0.1_0
*WRI1*	oil content	1738_736	10_3	1	0.6_0.4	1_0 (1)	0_0	0	0_0
*AGL11*	seed dormancy	587_253	8_1	0	1.4_0.4	1_0 (1)	1_0	0	0.2_0
*ATG5*	plant defense	1672_392	11_0	0	0.7_0	5_0 (1)	2_0	0	0.1_0
*FER*	disease resistance	1112_1112	7_7	1 + 2^d^	0.6_0.6	0_0 (0)	2_2	1	0.2_0.2
*GTR2*	glucosinolate transport	1200_1016	7_6	1^d^	0.6_0.6	1_3 (4)	0_0	0	0_0
*HAI2*	seed dormancy	1034_865	11_4	1^d^	1.1_0.5	1_1 (1)	0_0	0	0_0
*Total*		27,381_14,334	243_95	40		39_22 (21)	34_16	9	
*Mean*					0.9_0.8				0.14_0.16

^a^Values to the left of the underscore are for full length of the sequenced regions whereas values to the right of the underscore are only for the coding regions; ^b^ = applies only to the coding regions; ^c^ = values within parentheses refer to the total number of polymorphisms unique to *L. campestre*; ^d^ = polymorphism predicted as deleterious for protein function

### PCR and DNA sequencing

PCR mix for a 25 μl reaction volume contained 1× PCR buffer, 1.5 mM MgCl_2_, 0.3 mM dNTPs, 0.2 μM of each primer, 1 U Dream Taq polymerase (ThermoFisher Scientific) and 1 ng/μl DNA template. The conditions of PCR cycling were: 95 °C for 5 min; 35–40 cycles of 95 °C for 15 s, 52 to 60 °C, (depending on the primer-pair) for 15 s, and 72 °C for 40 s; and a final step of 72 °C for 10 min. PCR product clean-up and sequencing was performed at MWG Eurofins Genomics, Germany. DNA sequence data generated in this study have been deposited at the NCBI/GenBank and the accession numbers of the sequences are given in Additional file 2: Table S2.

### Sequence analyses

The diversity analyses are based on the 12 individuals (10 field cress and two CHe hybrids) from the RAD-sequencing and the 19 individuals (14 field cress, three *L. hirtum*, two *L. heterophyllum*) from the resequencing work. From the RAD-sequencing data, variants corresponding to each individual were generated using GATK’s HaplotypeCaller command (GATK version 3.5; a toolkit developed by Broad Institute). These variants were subsequently used to generate individual specific consensus sequences using GATK’s FastaAlternativeReferenceMaker command [48]. All chromatograms generated through resequencing of the 19 samples were visually evaluated using BioEdit version 7.0.5 [49], especially at the polymorphic sites to ensure that these sites were correctly scored. Then, multiple alignment for each of the 24 gene sequences of the 31 samples (19 samples from resequencing and 12 samples from RAD-sequencing) was performed using ClustalX version2.1 software [50], and then the sequences were manually edited using BioEdit version 7.0.5. Finally, variable sites in the forms of single nucleotide polymorphism (SNPs) and indels were tabulated. The same procedure was followed for the remaining six genes except that only sequences from 19 genotypes were used. The online PROVEAN tool (J. Craig Venter Institute) was used to predict the impact of the polymorphisms leading to a change in the amino acid sequence [51].

### Phylogenetic analyses

Two data sets were used for phylogenetic analyses of different genotypes of three *Lepidium* species and their hybrids. The first data set contains polymorphism only within the coding regions of the 30 genes whereas the second data set contain polymorphism within both the coding and non-coding regions of these genes. MEGA7 [52] software was used for the phylogenetic analyses through the application of methods that include Maximum Likelihood, Neighbor-Joining, Minimum Evolution, and Maximum Parsimony. However, most of the analyses produced highly similar results and hence trees generated using the Neighbor-Joining method [53] based on the evolutionary distances calculated according to Nei and Kumar [54] are presented in this paper. The bootstrap test [55] for the robustness of the tree branches was conducted based on 10,000 replications.

## Results

To identify polymorphisms that may contribute to domestication syndrome traits, perenniality and oil content and quality, partial sequences of 30 genes were analyzed in *Lepidium*. The protein coding sequences of field cress showed a high level of homology to *A. thaliana* with the sequence identity between *A. thaliana* and field cress ranging between 81 to 98% with a mean value of 89.2% (Table 2). A similar level of average sequence identity (89.3%) was observed when comparing field cress to *A. lyrata*, although differences were observed in more than 50% of the genes investigated. The comparative sequence analysis with additional Brassicaceae species showed that an average sequence identity between field cress and *Camelina sativa,* a minor oil crop grown in Europe and North America was 88.7%; *Capsella rubella* –a model plant used to study self-incompatibility in plant reproduction– showed similar level of sequence identity with field cress (88.5%). The commercially grown agricultural and horticultural crops *B. napus, B. oleracea* and *B. rapa* shared a slightly lower sequence identity with field cress with a mean of around 86% for the coding regions.

The alignment of partial gene sequences of field cress, CHe hybrids, *L. heterophyllum*, and *L. hirtum* revealed polymorphisms in all of the analyzed gene sequences. The distribution of polymorphisms for each gene sequence are listed in Table 3. Twenty-five of the gene sequences had SNPs or indels in the predicted coding regions and 19 of the gene sequences had at least one polymorphism that lead to an altered amino acid sequence. Polymorphisms that were predicted to be deleterious for protein function were recorded in six of the genes (*AGL16*, *FER, GTR2*, *HAI2, MAF5* and *SOC1*) (Table 3).

In total, 243 polymorphic sites were observed in 27,381 nucleotides long aligned sequences, which constitutes about 0.9% of the total analyzed nucleotides. The majority of the variations were single nucleotide substitutions but indels of variable lengths were also observed. Ninety-five of the polymorphisms were located in the predicted coding regions which constitute about 0.7% of the total length of the coding regions analyzed (14,334 nt). None of the sequences analyzed had mutations that would lead to an obvious severe defect in the protein, such as premature STOP codon or frame-shift indels. However, 40 of the identified polymorphisms were nonsynonymous and would result in an altered protein sequence. Seven of these polymorphisms were SNPs that were predicted to be deleterious for the protein function (Table 3). Moreover, 61 of the observed polymorphisms (39 in non-coding, and 22 in coding regions) were shown to be species specific, of which 21 were unique to field cress.

Within the field cress group of 24 individuals, 34 polymorphisms were observed in 13 different genes. Sixteen of the polymorphisms were located in the predicted coding regions of the *FAD2, FER*, *FRI, MAF2, MAF5, RPL, SHP1, SHP2* and *VRN1* genes. Nine of these polymorphisms are nonsynonymous but none were predicted to have a severe impact on the protein function, according to PROVEAN. The gene sequence of *SHP1* stands out by having most polymorphisms among the field cress individuals with six SNPs and two indels in the non-coding region and additionally three SNPs in the coding region. MAF2 has variable sites that accounted for 1.3% of the total nucleotides analyzed and 1.7% in coding region, which is significantly higher than the other genes in this study.

**Fig. 1:**
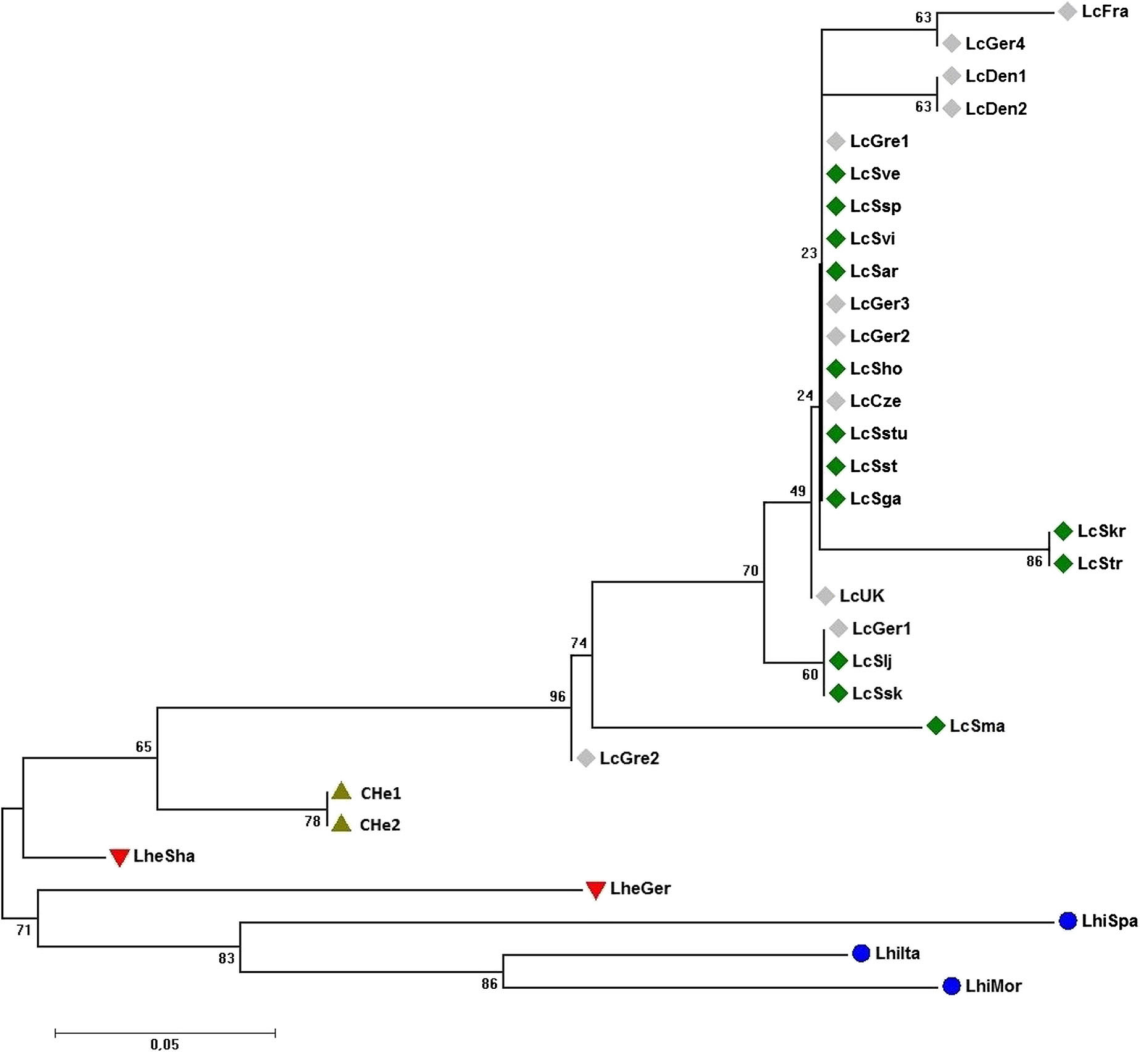
Cladogram showing the clustering pattern of individual genotypes representing field cress, CHe hybrids, *L. heterophyllum* and *L. hirtum* based on polymorphisms in coding regions. Green diamonds denote field cress accessions collected in Sweden and grey diamonds field cress from other parts of Europe. Green triangles = CHe hybrids, red triangles = *L. heterophyllum* and blue circles = *L*. *hirtum*. Numbers at the base of branches are bootstrap values

**Fig. 2:**
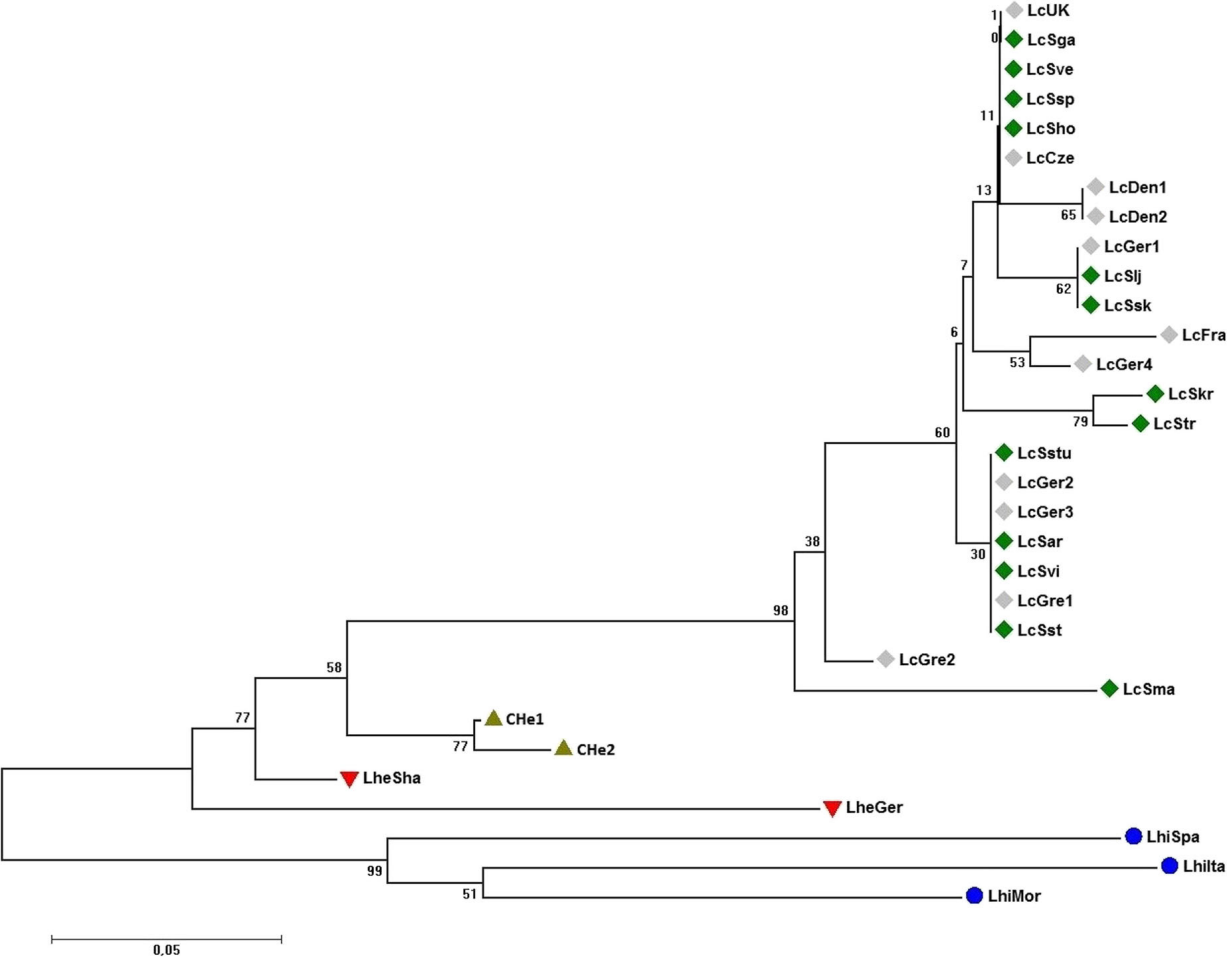
Cladogram showing the clustering pattern of individual genotypes representing field cress, CHe hybrids, *L. heterophyllum* and *L. hirtum* based on polymorphisms in both coding- and noncoding regions. Green diamonds denote field cress accessions collected in Sweden and grey diamonds field cress from other parts of Europe. Green triangles = CHe hybrids, red triangles = *L. heterophyllum* and blue circles = *L*. *hirtum*. Numbers at the base of branches are bootstrap values

Two different data sets were used for analysis of phylogenetic relationship between the individuals representing the three *Lepidium* species and CHe hybrids: a data set that contains only sequences of coding regions and another one that contains sequences of both coding and non-coding regions. Cladograms generated based on both data sets showed a formation of clearly separated branches representing the different species and the hybrids with a strong bootstrap support (Figs. 1 and 2). In both cases, field cress is shown to be most closely related to CHe hybrids and then to *L. heterophyllum*. The two cladograms slightly differ in the clade that comprised the field cress individuals although both showed a clear separation of genotypes LcGre2 and LcSma from the rest. Three genotypes (LcGer1, LcSlj and LcSsk), two of which represent Sweden and one represents Germany, formed a separate branch with a moderate level of bootstrap support in both data set. The use of combined data of coding and non-coding sequences produced a better resolution in this clade although some of the branches were supported by low bootstrap values, which is mainly because of low sequence divergence between the different genotypes of field cress.

## Discussion

### Sequence homology between various genes of field cress and other Brassicaceae species

Comparative genomics is a powerful tool that facilitates our understanding of the genetics of poorly studied plant species based on data from a closely related well-studied species. The close evolutionary relationship between field cress and the extensively studied model plant *A. thaliana* is advantageous and will be highly valuable for the domestication and further breeding of field cress*.* Alignments of the coding regions of the 30 genes confirm the close phylogenetic relationship between these two species. Comparing the mean sequence identity of the 30 genes suggests that *Lepidium* is more closely related to *Arabidopsis* than to *Brassica* (Table 2). The data also suggests closer evolutionary relationship of *Lepidium* with *Camelina* and *Capsella* than with *Brassica*. This finding correlates well with the recently released phylogeny of the Brassicaceae family using 113 nuclear DNA markers [56].

Many of the genes that are regulating flowering time and vernalization have more than 90% sequence identity to the *Arabidopsis* counterpart in this study (Table 2). Moreover, the top three genes with the highest level of polymorphisms per nucleotide in the coding regions (*FLC*, *FRI* and *MAF5*) also belong to this group of traits. However, in this group of genes, *FRI* stands out as one of the least conserved genes as it has a lower level of sequence homology to the other species it was compared with and has a relatively high level of variation in the coding region. Thus, this result suggests that *FRI* is evolving faster than most of the other genes analyzed in this study. Although *FRI* has been attributed to play a major role in response to vernalization and controlling flowering time in *A. thaliana*, the function of this gene in other species such as *A. lyrata* seems to be of less importance [57]. The role of *FRI* has not been completely elucidated for *B. rapa*, *B. napus* and *B. oleracea*, but it seems to have a less pronounced role in flowering time than in *A. thaliana* [58].

### Sequence variation in various genes between and within *Lepidium* species

Our genomic data showed that field cress is more closely related to *L. heterophyllum* than to *L. hirtum* (Figs. 1 and 2), which is in agreement with previous research based on chloroplast DNA analysis [59]. Field cress is a self-fertilizing plant, and as inbred populations they are expected to have low within-population variation. Hence, the clustering pattern of the genotypes (Figs. 1 and 2) clearly show lack of population differentiation in field cress according to geographical origin within Europe. Genotypes representing Sweden and other parts of Europe are distributed across the sub-branches within the field cress branch. This is similar to what was previously reported for *A. thaliana*; i.e., lacking a clear branching of different ecotypes, and only significant isolation by distance –such as separate continents– divides populations into distinct clusters [60]. However, the addition of considerably more DNA markers and screening of additional individuals have altered this hypothesis and clear global population structures seem to exist for *A. thaliana* [61]. In this study, only 24 field cress genotypes and short segments of the 30 genes were analyzed. Hence to reach a firm conclusion regarding the lack of population structure in European field cress populations, the use of additional polymorphic DNA markers and genotyping more individuals may be necessary.

The genotype LcGre2 differentiated from the main body of the field cress genotypes (Figs. 1 and 2). This genotype is of particular interest as it is more resistant to pod shattering and has a higher oil content compared to other field cress genotypes (unpublished data). It carries two mutations in the non-coding part of the *SHP1* gene which makes it different from all other field cress genotypes included in the present study, and which may be linked to mutation(s) in the coding region of this gene that confer shattering resistance. However, no mutations were found in the genes that encode for oil content and quality, and hence sequencing the full coding regions of these genes is needed to identify any useful mutations. One synonymous and one nonsynonymous mutation in the coding regions that contributed to the separation of this genotype from other field cress genotypes were found in the *FRI* gene, which regulates vernalization. No distinct vernalization requirement or winter hardiness were observed for the accession from which this genotype was developed. Multi-environment field trials are needed to determine the effect of these mutations. Similarly, LcSmar, a relatively high seed yielding genotype, was also clearly separated from the field cress cluster and have several mutations in the coding regions of *FRI*, *VRN1* and *MAF5*, which are genes regulating vernalization. This genotype has a unique SNP mutation in *FRI*, which is of particular interest. Hence, it will be very interesting to see how this accession perform in upcoming field trials held in the northern part of Sweden. In our ongoing crossbreeding experiments, hybridization of some field cress genotypes results in F_1_ hybrids with strong hybrid vigor, and some recombinant inbred lines derived from such hybrids maintain their vigor. On the other hand, some crosses result in weak F_1_ hybrids and subsequent generations. Hence, the clustering pattern of the 24 field cress genotypes in the present study facilitates the designing of an efficient crossbreeding scheme to identify best pairs of genotypes in terms of their combining ability.

Genetic variation was found in all analyzed genes but a higher level of polymorphisms was observed in the coding regions of genes regulating flowering time, vernalization and pod shattering. This result could be explained by the fact that individual plants displaying clear phenotypic differences for these traits were intentionally included in this study. A number of polymorphic sites among the three *Lepidium* species in the genes regulating oil content and quality was observed, but only two polymorphisms were observed within the field cress for these genes, whereof one is an indel in *FAD2* causing a deletion of a serine residue in two of the field cress genotypes. Field cress has generally a higher oil content, with average of about 20%, than the other two *Lepidium* species, with average about 15% (unpublished data). Hence further analysis of the variations within these genes followed by well-thought crossbreeding may lead to an increase in oil content in field cress.

Flowering time is one of the most important agronomic traits and is preceded by a vernalization period in perennial, biennial and winter type annual plants. Timing of flowering at the most favorable conditions is important to optimize the seed production in a crop. Vernalization ensures that the frost-sensitive transition from vegetative to reproductive growth to occur at milder temperatures. In this study, 13 gene sequences related to flowering time and vernalization were analyzed for natural variation and compared with other closely related species in terms of sequence homology. This group of genes show the highest level of variation in the sequenced coding regions, compared to the genes coding for other traits considered in this study. *FLC*, *MAF5, MAF2* and *FRI* come on top as per their polymorphisms per nucleotide in the protein coding regions (Table 3), suggesting that they are the fastest evolving genes, in that order, when compared to other genes. *Lepidium FRI* also has a lower sequence homology to *A. thaliana FRI* (*AtFRI*), compared to other genes in this study except *ATG5*, which has the same level of homology. In this gene, a high degree of variation between the biennial species (field cress) and the perennial species (*L. heterophyllum* and *L. hirtum*) was observed, thus the genetic variation between the two groups could be linked to perenniality.

The transition between vegetative and reproductive phase is mainly controlled by *FLC* and its positive regulator FRI. In fact, polymorphisms in these genes account for most of the natural variation found in flowering time in *A. thaliana* [62]. Several naturally occurring non-functional *AtFRI* alleles have been reported and the majority of them derive from deletions in the protein coding region [17, 62]. Six nonsynonymous SNPs were observed in the *FRI* sequence among the three *Lepidium* species but no polymorphisms resulting in a deletion of amino acid sequence was found. Our data shows a high level of polymorphisms in the *FRI* gene, both in coding and non-coding sequences. This finding may indicate that *FRI* does not have a dominant role in the biennial field cress as it has been shown that *FRI* has a redundant function in *A. lyrata*, which has a perennial life cycle [57]. As mentioned above, the sequence homology for the coding sequences of the *FRI* gene is higher between field cress and *A. lyrata* (84%) than between field cress and *A. thaliana* (81%). However, the homology is basically the same for both pairs at the protein level.

Multiple studies in *A. thaliana* failed to find any nonsynonymous polymorphisms in the *AtFLC* gene. Of the few polymorphisms found, the majority were located in the first intron [63]. One of these polymorphisms separate *AtFLC* alleles into two distinct haplogroups that flowers at significantly different times in a null-*FRI* background [64]. The low abundance of polymorphisms in the coding region of *AtFLC* is in line with our findings in the partial sequences of field cress *FLC* gene, as no variation was detected among the field cress genotypes. However, among the three *Lepidium* species *FLC* had one of the highest number of polymorphisms per nucleotides, both in coding and non-coding regions. Hence, although this gene seems to be conserved within the field cress, a high level of diversity between closely related *Lepidium* species is apparent. On the other hand, the sequence identity between field cress and several Brassicaceae species was overall high with a sequence identity of 88 to 92% for the coding sequence of this gene (Table 2). The *FLC* gene in *A. lyrata* has been tandemly duplicated and one of the paralogs are having a more pronounced effect on vernalization than the other [65, 66]. Two separate partial sequences of the field cress *FLC* gene were sequenced in this project, targeting the 5′ and 3′ regions of the coding sequence. A nucleotide BLAST search shows that the 5′ region is more similar to the *FLC2* gene in *A. lyrata*, whilst the 3′ region is more homologous to the *FLC1* gene. Hence it cannot be ruled out that there are more than one copy of the *FLC* gene in field cress, and that we have targeted both paralogs.

Downregulation of *FLC* promotes floral development while low levels of *FLC* are maintained after cold treatment by *VRN1* and *VRN2* in *Arabidopsis*, which are serving as a molecular memory of the vernalization [21, 67]. Interestingly, compared to other genes included in this study, an extremely high level of polymorphisms has been reported for *AtVRN2*, over 140 mutations in the UTR and introns, and 55 in the coding region, resulting in 4% of the nucleotides in the coding region being polymorphic [68]. Even though 536 nt long coding sequences of *Lepidium VNR2* were analyzed, only one SNPs was observed among the three *Lepidium* species. Thus, less than 0.2% of the nucleotides in this region are variable. When delimiting the observed polymorphisms of *AtVRN2* to the corresponding region to this 536 nt long segment, and recalculating based on this, it seems as this region is less variable than other parts of the gene as only 2% of the nucleotides are polymorphic in *A. thaliana*. However, that is still ten times more variable than in *Lepidium*. Hence, the *VRN2* gene appears to be more conserved within the *Lepidium* species than in *A. thaliana*. Moreover, very few polymorphisms were observed in *VRN1* among the *Lepidium* species in this study, but this is more in line with what have been reported for the *Arabidopsis* ortholog.

*SOC1* is an integrator of the floral pathway under direct repression of *FLC*. *FUL* is a *MADS* box gene that operates downstream of *SOC1* in the same pathway. Perenniality is normally considered to be a complex trait. However, a double null mutant of *FUL* and *SOC1* could transform an annual *Arabidopsis* into something that is reminiscent of a perennial plant [24]. While the controls senesced after flowering, the *FUL/SOC1* mutant plant returned to vegetative stage and eventually developed into a highly-branched shrub with woody stems, which are traits associated with perennials. Moreover, expression levels of *FUL* and *SOC1* are significantly reduced in the *A. thaliana* ecotype SY-0, which has a similar morphology as the *FUL/SOC1* mutant [69]. This indicate that *SOC1* and *FUL* genes may be important in controlling longevity in a plant. Only a single nonsynonymous polymorphisms have been reported for *AtFUL* and none for *AtSOC1* [68]. Such a result is comparable with our findings in the three *Lepidium* species, in which we only found one missense mutation that differentiates *L. hirtum* ssp. *atlanticum* from field cress, *L. heterophyllum* and the other two subspecies of *L. hirtum* in SOC1 and none in *FUL*. Hence, this mutation cannot be attributed to perenniality trait. *Lepidium FUL* and *SOC1* also share a very high sequence homology with *AtFUL* and *AtSOC1*, thus indicating that these genes are highly conserved. The difference in regulation of these genes, rather than polymorphism within the coding gene sequence, is therefore likely to be the cause of the observed phenotypic variation in the double mutant of *FUL* and *SOC1*, and the difference in longevity for field cress versus its perennial relatives.

For wild plants, it is generally advantageous to disperse all seeds to increase the survival chances of future generations. In contrast, domesticated plants are more resistant against shattering which ensures farmers’ maximum seed harvest. Resistance to pod shattering and seed dispersal is an important agronomic trait as shattering can result in serious yield losses. In *Arabidopsis*, the seed-containing fruit is an ovary composed of three main tissue types: the valve, the replum and the valve margin. The valve margin separates the valve from the replum and also referred to as the dehiscence zone. Upon fruit dehiscence, the valve margin detaches from the replum and the seeds are released. The two transcription factor genes (*ALC* and *IND*) are required for valve margin formation and are promoted by MADS box genes *SHP1* and *SHP2* [31–33]. *ALC*, *IND*, *SHP1* and *SHP2* are collectively known as the valve identity genes. *FUL* and *RPL* genes are expressed in the valve and replum respectively and negatively regulates the valve identity genes to ensure that these genes are expressed in the proper tissue [30, 34]. Many aspects of fruit dehiscence have been studied in field cress. There is a high degree of conservation of the fruit development pathways between field cress and *Arabidopsis* and expression levels of the field cress valve identity genes resembles those of *A. thaliana* [12, 70] Many of the genes involved in seed shattering displayed a relatively high level of variation per nucleotides in this study. This mirrors the phenotypic variation in this particular trait among the field cress genotypes in the present study (unpublished data).

The *SHP1*, *ADPG2*, *IND* pod shattering genes analyzed in *Lepidium* also had a higher level of variation in the coding region per nucleotides (1.1–1.5%) than the average variation for all genes obtained in this study. The sequences of *ADPG2* and *IND* also are less similar to the *A. thaliana* counterparts (84 and 85%, respectively), thus indicating that these genes are not as conserved as most other genes in this study. A nonsynonymous mutation in *RPL* divided the field cress individuals into two groups, where the individuals having one of the alleles display medium to high level of pod shattering while those having the other allele have a lower tendency towards shattering. *RPL* has proven to be an important regulator of this trait in *B. rapa*, as well as in *Oryza sativa* as a single point mutation in the regulatory sequence of this gene seems to be responsible for seed shattering resistance [71, 72]. The shattering type allele is present in *A. thaliana* as well as in field cress which is naturally highly shattering [73].

Seeds with a high oil content and a desirable oil composition is essential for developing an economically viable oilseed crop. Partial sequences of *Lepidium* that are orthologous to *Arabidopsis FAD2*, *FAE1*, *WRI1*, *TAG1* and *KCS8* which are all involved in fatty acid biosynthesis were analyzed in this study. Much of the natural variation in seed oil composition in *Arabidopsis* has been attributed to *FAD2* and *FAE1* [74]. Indeed, downregulation of *FAD2* and *FAE1* in field cress also results in altered oil composition, drastically increasing the proportion of desirable oleic acid in the oil [75]. All the five genes shared high homology to the *Arabidopsis* orthologs and nonsynonymous polymorphisms were observed in *FAD2*, *KCS8* and *WRI1*. One of the nonsynonymous SNPs was located in the start codon of the predicted amino acid sequence of *KCS8*. This allelic difference only results in a five amino-acid shortening of the protein as an additional in-frame start codon is positioned close by. Moreover, this shortening was not predicted to have a severe effect on the final protein according to PROVEAN. In *FAE1* there are two SNPs in the coding region differentiating field cress from the *L. heterophyllum* and *L. hirtum*. As field cress has a relatively higher oil content than the other two *Lepidium* species these polymorphism could be important markers for this trait.

The immediate challenges of breeding field cress into an economically viable new perennial oil crop includes countering unstable longevity and weak shattering resistance, and increasing the oil content [9, 73]. In this study, we have characterized partial sequences of the genes regulating these traits. The continuation of the work includes identifying the full gene sequences and characterize them. In future research, it would be necessary to include cis-regulatory elements as there are examples of important polymorphisms in these regions responsible for the traits that are associated with the “domestication syndrome” [71]. Linkage mapping, and detailed quantitative trait loci maps, which are in progress at the moment, will also be needed to advance the breeding of field cress. Furthermore, the agronomic practice for perennial crops is not fully developed, which is also the case for field cress.

With these findings, we have begun to unravel the genomics of field cress and the results will be the foundation for the future breeding strategy of this potential oilseed crop. Moreover, the outcome of this study has contributed to the overall understanding of *Lepidium* genome evolution.

## Conclusion

This study is the first performed on *Lepidium* genes with the purpose of improving field cress as a future oilseed crop. It has revealed significant variation among the *Lepidium* species within the partial sequences of 30 genes known to regulate traits such as flowering, perenniality, pod shattering and more. The phylogenetic relationship demonstrated between the three *Lepidium* species in this study can guide the development of interspecific hybridization to advance the domestication process of field cress.

## Additional files

Additional file 1: **Table S1.** Genomic DNA sequences. (XLSX 23 kb)

Additional file 2: **Table S2.** List of genes, accession numbers and primer sequences. (DOCX 36 kb)

## Abbreviations

*ADPG1*/2: *ARABIDOPSIS DEHISCENCE ZONE POLYGALACTURONASE1/2* gene
*AGL11*: *AGAMOUS-LIKE 11* gene
*AGL16*: *AGAMOUS LIKE 16* gene
*AGL6*: *AGAMOUS LIKE 6* gene
*ALC*: *ALCATRAZ* gene
*AP2*: APETALA2 gene
At: *Arabidopsis thaliana*
*ATG5*: *AUTOPHAGY RELATED 5* gene
BLAST: Basic local alignment search tool
CHe hybrids: *Lepidium campestre*- *Lepidium heterophyllum* hybrids
*FAD2*: *FATTY ACID DESATURASE 2* gene
*FAE1*: *FATTY ACID ELONGATION 1* gene
*FER*: *FERONIA* gene
*FLC*: *FLOWERING LOCUS C* gene
*FRI*: *FRIGIDA* gene
*FUL*: *FRUITFULL* gene
*HAI2*: *HIGHLY ABA-INDUCED PP2C PROTEIN 2* gene
*IND*: *INDEHISCENT* gene
*KCS8*: *3-KETOACYL-COA-SYNTHASE 8* gene
Lc: *Lepidium campestre*
MADS box: *MINICHROMOSOME MAINTENANCE1, AGAMOUS, DEFICIENS and SERUM RESPONSE FACTOR* box
*MAF2–5*: *MADS AFFECTING FLOWERING 2–5* genes
*NAC012*: *NAC DOMAIN CONTAINING PROTEIN 12* gene
Nt: nucleotides
RAD: Restriction site associated DNA
*RPL*: *REPLUMLESS* gene
*SHP1/2*: *SHATTERPROOF1/2* genes
SNP: Single nucleotide polymorphism
*SOC1*: *SUPRESSOR OF OVEREXPRESSION OF CO1* gene
*TAG1*: *ACYL-COA: DIACYLGLYCEROL ACYLTRANSFERASE 1* gene
*VIN3*: *VERNALIZATION INSENSITIVE 3* gene
*VRN1*/2: *REDUCED VERNALIZATION RESPONSE1/2* gene
*WRI1*: *WRINKLED 1* gene
