# Supplementary material for: Identification of genes regulating traits targeted for domestication of field cress (Lepidium campestre) as a biennial and perennial oilseed crop
Source: BMC Genet. 2018 May 29;19:36. doi: 10.1186/s12863-018-0624-9 (PMC5975587; doi:10.1186/s12863-018-0624-9)
Supplement: Supplementary file 2 — Table S2. List of genes, accession numbers and primer sequences. (DOCX 36 kb) [file 12863_2018_624_MOESM2_ESM.docx]

**Table S2.** Genes with respective accession number used in this study and primer sequences for targeted sequences in *L. campestre*. Two separate regions of the *FLC* gene were targeted (*FLC*-3’ and *FLC*-5’).

| Gene | Accession number | Primer forward 5'-3' | Primer reverse 5'-3' |
| --- | --- | --- | --- |
| *ADPG1* | KY502254 | TGATTGTATATCCATTGAGGATGG | TGAAATTTAATGTTCTTGGCAGTT |
| *ADPG2* | KY613954 | CTGCGGTTCAAGTGAAGAATG | GCATTGAGGCAAAACAGCTC |
| *AGL6* | KY502259 | TCTTGAGAAGAAGAAAATCAGAATC | ATGTACGGACCGACCCATTA |
| *AGL11* | KY502258 | TCTGATGGGAGACTCTTTGAG | CATTTGATGATGGTGTTGTTGA |
| *AGL16* | KY659391 | AATCGTTTGATGGATTGAAGAGA | TTCATTTCAAGAACCAACCAAG |
| *ALC* | KY613949 | GAGCAAGATCAACGAGAAAATG | GACTCCTGTGTTTGGCAGGT |
| *AP2* | KY613946 | AACGGGTCGGATCTTTCTCT | ACTCCACGGAATTTTATAGCTG |
| *ATG5* | KY502256 | ATTTACCACCCACGAAAACG | ATCGGCCTGTTATGTTGCTC |
| *FAD2* | KY613948 | AACGCACTTTCCATTTTTGG | CCAGGAGAAGTAAGGGACGA |
| *FAE1* | KY613947 | CCAACTCCTTCGCTATCTGC | ACTTTTCGCTCAACGGAAGA |
| *FER* | KY502260 | TAAGGCTGTTGTTGCAGGTG | TGTTGGGTTTAAGGCTGGTC |
| *FLC*-3' | KY502269 | TACTTGAACTTGTGGATAGGTT | TCAACAAGCTTCAACAT |
| *FLC*-5' | KY502268 | TAGAAATCAAGCGAATTGAGAAC | ATTTGGTTATCTCATGTATCT |
| *FRI* | KY502255 | TCTCTGCAAGACAAGTTTCGCT | GTCGAACAGCCAAGCCAATG |
| *FUL* | KY613951 | AGGAATTTTATGGGGGAAGA | TTTCATTCAATCCCATTTCAA |
| *GTR2* | KY502262 | CTGGCTGCAACTTGTCTTCA | TGGGTCTTGGGTTTCTTGTC |
| *HAI2* | KY502257 | CGGCGATTCTAGAGCAGTTC | ACCCCACCACTTCCTCTTTC |
| *IND* | KY631692 | CCCAAAGAAGCATGATGGAG | GGAGGTCCAAGGTGAGAGTG |
| *KCS8* | KY502253 | CTTAGAACGTTCCGGTCTCG | CAATCCGGAACAAGCAGTTT |
| *MAF2* | KY659392 | TCCGGCGAGTAATACAGACA | CGATGAACAAAACAAGAACATCA |
| *MAF5* | KY502264 | CGCATCGTTTAGTGGTCTTT | CCTATCACCGGAGGAGAAGC |
| *NAC012* | KY502263 | ATCTTGATGTCATTCGTGAAGTTG | GTCGAGGCGATACTCATGC |
| *RPL* | KY613950 | CTCTCGCTGATTCCGATTTC | TCGTCGAGCATGGAGATGAG |
| *SHP1* | KY613952 | GTATTTCAGTTAACGATTGGTTTGG | GCTGCAACTCCATTTCCTACA |
| *SHP2* | KY613953 | CATTCTTGGTGAATCTCTTGGTT | GTTGCAGAGGTGGTTGGTC |
| *SOC1* | KY502261 | CAGTATGCAAGATACCATAGATCG | CTATGCCTTCTCCCAAGAGTTTAC |
| *TAG1* | KY659389 | TCTAAACCTCGCGTCTTTGG | AGCGATGTTTTAGGGAGGAA |
| *WRI1* | KY659390 | CGAGCCAAGAAGGCTAAGAA | TGGTGTCAGGTCCCCAGTA |
| *VIN3* | KY502267 | TTGCTTGTAACACCCTGCAA | AAAGCTTGAGGCAAATTCCA |
| *VRN1* | KY502265 | AGAAAAACGCCGATCCAGGT | TGATCATGAGCAGAGAAGACCA |
| *VRN2* | KY502266 | CCCTGCAAAGACACAAGACA | CAATCGTGTTTTGGTTAGGTCA |
